# Dataset of urinary metabolites measured by ^1^H NMR analysis of normal human urine

**DOI:** 10.1016/j.dib.2016.11.101

**Published:** 2016-12-07

**Authors:** Marc Cassiède, Sindhu Nair, Meghan Dueck, James Mino, Ryan McKay, Pascal Mercier, Bernadette Quémerais, Paige Lacy

**Affiliations:** a Pulmonary Research Group, Department of Medicine, University of Alberta, Edmonton, AB, Canada; b Department of Chemistry, University of Alberta, Edmonton, AB, Canada; c National High Field NMR Centre (NANUC), University of Alberta, Edmonton, AB, Canada

**Keywords:** Metabolomics, Validation, Nuclear magnetic resonance

## Abstract

The data in this article are related to the research entitled, “Assessment of ^1^H NMR-based metabolomics analysis for normalization of urinary metals against creatinine” (M. Cassiède, S. Nair, M. Dueck, J. Mino, R. McKay, P. Mercier, B. Quémerais, P. Lacy, 2016) [1]. This article describes the analysis of urinary metabolites in normal, healthy individuals by ^1^H NMR-based metabolomics. NMR spectra of urine samples typically contain hundreds of peaks that must be carefully screened for reproducibility and detectability. An important requirement in the screening of appropriate urinary metabolites is to ensure that they are reproducibly detected. In our study, we applied the peak profiles of 151 known urinary metabolites to 10 normal human urine samples and found that 50 metabolites were reproducibly measured between 600 and 700 MHz magnets in the same samples. The data set has been made publicly available to enable critical or extended analysis.

**Specifications Table**

**Table:** 

Subject area	*Clinical Chemistry*
More specific subject area	*Metabolomics*
Type of data	*Table*
How data was acquired	*NMR spectroscopy using Varian 600* *MHz and Agilent 700* *MHz magnets*
Data format	*Analyzed and ranked according to correlation coefficient (R*^*2*^*)*
Experimental factors	*Urine samples from 10 normal, healthy human subjects were collected and measured by NMR using two different magnets (600 and 700 MHz) to validate quantities of detected urinary metabolites*
Experimental features	*Normal human urine samples from fasting subjects were supplemented with DSS internal standard, had their pH adjusted to 7.0±0.25, and were measured by NMR spectroscopy*
Data source location	*Pulmonary Research Group, Edmonton, Alberta, Canada*
Data accessibility	*Not applicable*

**Value of the data**

• NMR spectroscopy is rapidly becoming a major tool in human urine analysis for a range of applications. At present, there are few studies testing the validity of NMR-based approaches in broad-spectrum metabolomics analysis of human urine samples.

• The data shows that urinary metabolites measured by NMR analysis should be carefully assessed for reproducibility to validate the individual metabolites that are detected.
• One method of assessing reproducibility is to measure the same samples on two different NMR magnets and correlate the metabolite measurements between the two systems.
• These findings have broad implications for measurement of metabolites in human urine samples.

## 1 Data

Urinary metabolites detected in 10 normal, healthy human urine samples from fasting subjects were collected from two different ^1^H NMR magnets (600 and 700 MHz). Pearson correlation coefficient was estimated for 151 metabolites that were selected based on their known presence in human urine samples [2], [3]. A total of 57 metabolites (38%) were detected on both 600 and 700 MHz systems that had a strong *R*^2^ of >0.7, listed in Supplementary Table 1. A bias of >0.5 was present in 7 additional samples, based on slope linearity of each metabolite, with a final total of 50 metabolites (33%) could be reproducibly detected on the 2 different NMR magnets within the same 10 urine samples.

## 2 Experimental design, materials and methods

### 2.1 Human urine sample collection

Urine samples from 10 fasting human subjects (2 males, 8 females, 20–53 yr) were collected and centrifuged at 600 g on a standard refrigerated benchtop centrifuge at 4 °C for 10 min to remove precipitates. Supernatants were vortexed and had DSS containing internal standard added followed by pH adjustment to 7.0±0.25 to ensure uniformity in pH values across the samples [1].

### 2.2 Measurement of chemical shifts of urinary metabolites by ^1^H NMR analysis

Samples of urine were transferred into 3 mm (for 700 MHz magnet) or 5 mm (for 600 MHz) NMR tubes and spectra were collected as described [1].

### 2.3 NMR spectral and statistical analysis

Peak-fitting on the resulting spectra was done using a computer algorithm associated with Chenomx NMR Suite 8.0 software to generate concentrations of detected urinary metabolites (Chenomx, Edmonton, AB, Canada). Linear regression analysis of the resulting urinary metabolites from 600 and 700 MHz magnets were compared using GraphPad Prism 6 software (GraphPad Software, La Jolla, CA) [1].
