# Supplementary material for: Dataset of urinary metabolites measured by 1H NMR analysis of normal human urine
Source: Data Brief. 2016 Dec 7;10:227–9. doi: 10.1016/j.dib.2016.11.101 (PMC5155042; doi:10.1016/j.dib.2016.11.101)
Supplement: Supplementary file 1 — Supplementary material [file mmc1.pdf]

Conflicts of Interest Statement

Manuscript title: Dataset of urinary metabolites measured by 1H NMR analysis of normal human urine

The authors whose names are listed immediately below certify that they have NO affiliations with or involvement in any organization or entity with any financial interest (such as honoraria; educational grants; participation in speakers' bureaus; membership, employment, consultancies, stock ownership, or other equity interest; and expert testimony or patent-licensing arrangements), or non-financial interest (such as personal or professional relationships, affiliations, knowledge or beliefs) in the subject matter or materials discussed in this manuscript.

Author names:

Marc Cassiede, Sindhu Nair, Meghan Dueck, James Mino, Ryan McKay, Pascal Mercier, Bernadette Quemerais, Paige Lacy

The authors whose names are listed immediately below report the following details of affiliation or involvement in an organization or entity with a financial or non-financial interest in the subject matter or materials discussed in this manuscript. Please specify the nature of the conflict on a separate sheet of paper if the space below is inadequate.

Author names:

This statement is signed by all the authors to indicate agreement that the above information is true and correct (*a photocopy of this form may be used if there are more than 10 authors*):

Author's name (typed)	Author's signature	Date
Paige Lacy	[illegible]	November 24, 2016
Marc Cassiede	[illegible]	November 24, 2016
Sindhu Nair	[illegible] for SN	November 24, 2016
Meghan Dueck	Meghan Dueck	Nov. 24 2016
James Mino		
Ryan McKay		
Pascal Mercier		
Bernadette Quemerais	[illegible]	November 24, 2016

This statement is signed by all the authors to indicate agreement that the above information is true and correct (*a photocopy of this form may be used if there are more than 10 authors*):

Author's name (typed)	Author's signature	Date
Paige Lacy		November 24, 2016
Marc Cassiede		
Sindhu Nair		
Meghan Dueck		
James Mino		Nov. 24, 2016
Ryan McKay		
Pascal Mercier		
Bernadette Quemerais		

This statement is signed by all the authors to indicate agreement that the above information is true and correct (*a photocopy of this form may be used if there are more than 10 authors*):

Author's name (typed)	Author's signature	Date
Paige Lacy		November 24, 2016
Marc Cassiede		
Sindhu Nair		
Meghan Dueck		
James Mino		
Ryan McKay		Nov. 24, 2016
Pascal Mercier		
Bernadette Quemerais		

This statement is signed by all the authors to indicate agreement that the above information is true and correct (*a photocopy of this form may be used if there are more than 10 authors*):

Author's name (typed)	Author's signature	Date
Paige Lacy		November 24, 2016
Marc Cassiede		
Sindhu Nair		
Meghan Dueck		
James Mino		
Ryan McKay		
Pascal Mercier	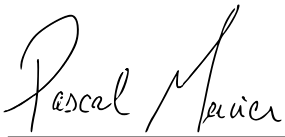	
Bernadette Quemerais		
