# Supplementary material for: Dataset of urinary metabolites measured by 1H NMR analysis of normal human urine
Source: Data Brief. 2016 Dec 7;10:227–9. doi: 10.1016/j.dib.2016.11.101 (PMC5155042; doi:10.1016/j.dib.2016.11.101)
Supplement: Supplementary file 2 — Supplementary material [file mmc2.docx]

**Supplementary Table 1.** Urinary metabolites identified by NMR with their associated correlation values between 600 and 700 MHz magnets.

| **Number** | **Compound Name** | **R^2^ value** |
| --- | --- | --- |

1 Glycine **0.9997**

2 Hippurate **0.9996**

3 Citrate **0.9988**

4 Dimethylamine **0.9985**

5 Creatinine **0.9973**

6 *U233 **0.9972**

7 *Uarm1 **0.9938**

8 Trigonelline **0.9935**

9 Formate **0.9922**

10 3- Hydroxyphenylacetate **0.9897**

11 Alanine **0.9893**

12 *U433 **0.9884**

13 *Uarm2 **0.9870**

14 2- Hydroxyisobutyrate **0.9795**

15 *U11 **0.9751**

16 *U185 **0.9747**

17 *U122 **0.9694**

18 Trimethylamine N-oxide **0.9674**

19 Acetone **0.9569**

20 Acetaminophen **0.9494**

21 5- Aminolevulinate 0.9389

22 3- Hydroxyisovalerate **0.9368**

23 4- Hydroxybenzoate **0.9356**

24 *U122triplet **0.9352**

25 3-Hydroxyisobutyrate **0.9075**

26 *U362 0.8820

27 3- Aminoisobutyrate **0.8771**

28 Serotonin 0.8769

29 Valine **0.8749**

30 2- Oxoglutarate **0.8720**

31 Carnitine **0.8700**

32 Propylene glycol **0.8689**

33 Glutamine **0.8538**

34 *U144 **0.8498**

35 Hypoxanthine **0.8496**

36 Histidine **0.8494**

37 Glycolate **0.8426**

38 Sucrose **0.8383**

39 *U380large **0.8381**

40 1- Methylnicotinamide **0.8353**

41 *U14 **0.8346**

42 Azelate **0.8343**

| **Number** | **Compound Name** | **R^2^ value** |
| --- | --- | --- |

43 Methanol **0.8054**

44 *U43 0.7984

45 2-Aminoadipate 0.7928

46 Creatine **0.7918**

47 O-Acetylcarnitine **0.7791**

48 Glucose-6-phosphate 0.7751

49 N-Acetylornithine **0.7746**

50 N-Acetylglutamine **0.7721**

51 3-Hydroxymandelate **0.7663**

52 *U361 **0.7580**

53 5-Hydroxytryptophan **0.7453**

54 Galacterate **0.7452**

55 Ethanolamine **0.7381**

56 Phenylalanine 0.7332

57 Pseudouridine **0.7123**

Bold represents metabolites with low bias (< 0.5) between 600 and 700 MHz magnets.

* Unknown urinary metabolites not found in the Chenomx 600 MHz NMR library.
